# Comparative genomic analyses provide new insights into evolutionary history and conservation genomics of gorillas

**DOI:** 10.1186/s12862-023-02195-x

**Published:** 2024-01-26

**Authors:** Tom van der Valk, Axel Jensen, Damien Caillaud, Katerina Guschanski

**Affiliations:** 1 Centre for Palaeogenetics, Stockholm, Sweden; 2 Department of Bioinformatics and Genetics, Swedish Museum of Natural History, Stockholm, Sweden; 3 SciLifeLab, Stockholm, Sweden; 4 Department of Zoology, Stockholm University, Stockholm, Sweden; 5 Department of Ecology and Genetics, Animal Ecology, Uppsala University, Uppsala, Sweden; 6 Department of Anthropology, University of CA – Davis, Davis, California USA; 7 Institute of Ecology and Evolution, School of Biological Sciences, University of Edinburgh, Edinburgh, UK

**Keywords:** Inbreeding, Gene flow, Genetic diversity, Local adaptation

## Abstract

Genome sequencing is a powerful tool to understand species evolutionary history, uncover genes under selection, which could be informative of local adaptation, and infer measures of genetic diversity, inbreeding and mutational load that could be used to inform conservation efforts. Gorillas, critically endangered primates, have received considerable attention and with the recently sequenced Bwindi mountain gorilla population, genomic data is now available from all gorilla subspecies and both mountain gorilla populations. Here, we reanalysed this rich dataset with a focus on evolutionary history, local adaptation and genomic parameters relevant for conservation. We estimate a recent split between western and eastern gorillas of 150,000–180,000 years ago, with gene flow around 20,000 years ago, primarily between the Cross River and Grauer’s gorilla subspecies. This gene flow event likely obscures evolutionary relationships within eastern gorillas: after excluding putatively introgressed genomic regions, we uncover a sister relationship between Virunga mountain gorillas and Grauer’s gorillas to the exclusion of Bwindi mountain gorillas. This makes mountain gorillas paraphyletic. Eastern gorillas are less genetically diverse and more inbred than western gorillas, yet we detected lower genetic load in the eastern species. Analyses of indels fit remarkably well with differences in genetic diversity across gorilla taxa as recovered with nucleotide diversity measures. We also identified genes under selection and unique gene variants specific for each gorilla subspecies, encoding, among others, traits involved in immunity, diet, muscular development, hair morphology and behavior. The presence of this functional variation suggests that the subspecies may be locally adapted. In conclusion, using extensive genomic resources we provide a comprehensive overview of gorilla genomic diversity, including a so-far understudied Bwindi mountain gorilla population, identify putative genes involved in local adaptation, and detect population-specific gene flow across gorilla species.

**Supplementary Information:**

The online version contains supplementary material available at 10.1186/s12862-023-02195-x.

## Introduction

The conservation of biodiversity is a critical global challenge, and genetic approaches are becoming increasingly important for this endeavor [79]. Genomics has revolutionized our ability to study wild species, providing unprecedented observations into population structure, diversity, evolutionary history and local adaptation [79]. These insights have important implications for conservation, including the identification of vulnerable populations, the design of effective conservation plans, and the development of strategies for the management of threatened or endangered species [79].

**Fig. 1:**
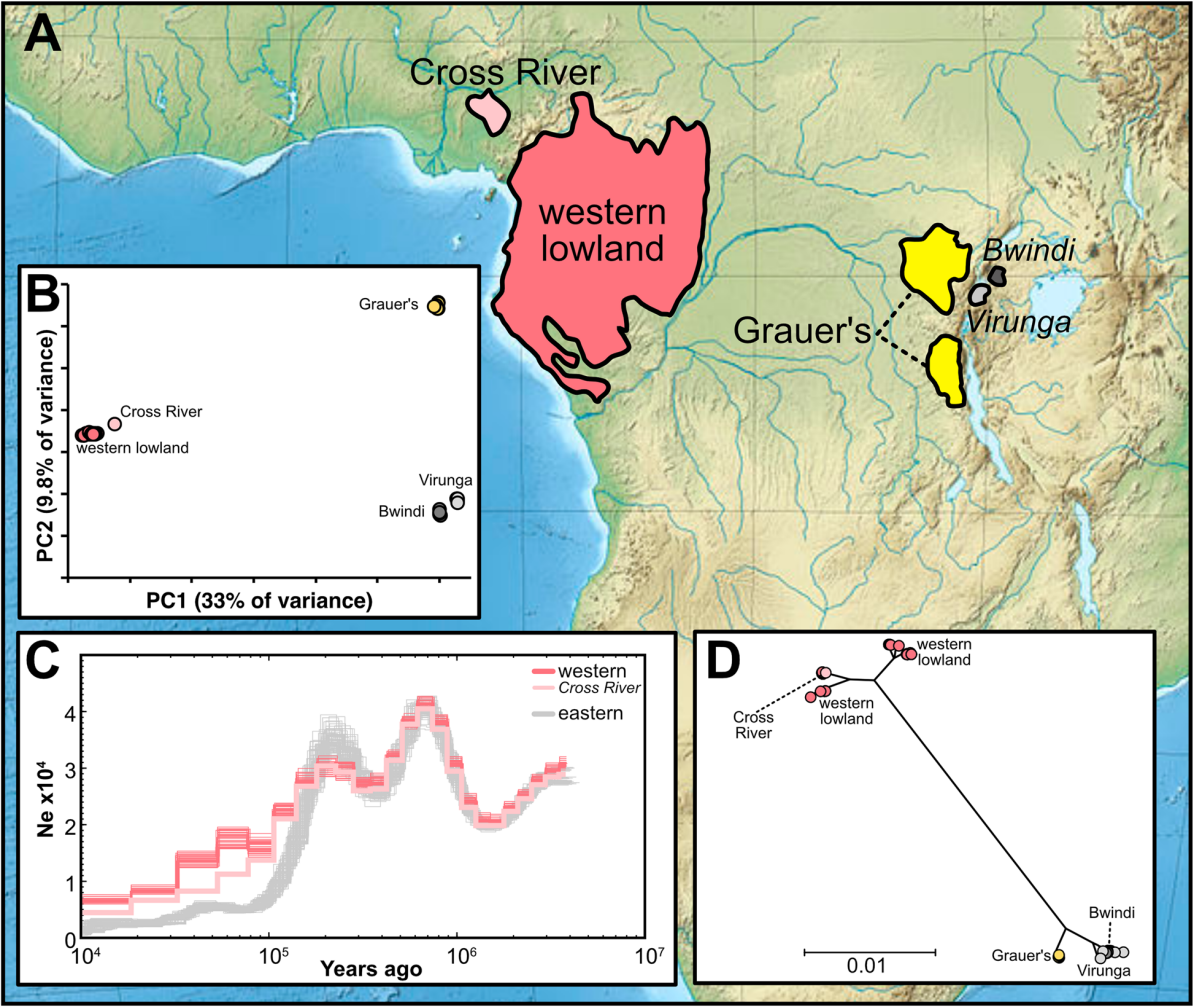
**A** Geographic distribution of the gorilla subspecies [38–41]. **B** Principal component analysis based on all autosomal SNPs. **C** Demographic trajectories of gorillas over the last two million years based on beta-PSMC analyses. **D** Neighbour-joining mitochondrial tree. Distance measure depicts substitutions per site

Gorillas are one of the most iconic and endangered primates and since the early days of DNA sequencing, genetic approaches have been used to study gorilla population structure, relatedness, and gene flow, starting from short D-loop and microsatellite analyses in the early 1990s to the first whole genome sequences in 2010s [23, 65, 89]. Two gorilla species are recognized: the relatively abundant western gorillas (*Gorilla gorilla*) with an estimated census population size of around 360,000 individuals and the rarer eastern gorillas (*G. beringei*) with possibly fewer than 10,000 individuals currently remaining [27, 75, 36]. The two species are geographically separated by the Congo River basin, genetically, morphologically and ecologically distinct from each other, and further classified into two subspecies each: In the west of Central Africa are the western lowland (*G. g. gorilla*) and Cross River gorillas (*G. g. diehli*), and in East Africa the Grauer’s (*G. b. graueri*) and mountain (*G. b. beringei*) gorillas (Fig. 1A, B). All four gorilla subspecies experienced population declines for at least the past 100,000 years, with the declines being most pronounced in the two eastern subspecies [90] (Fig. 1C). In recent times, habitat loss, poaching, and disease outbreaks have further decimated the gorilla populations, leading to reduction in genetic diversity and increase in inbreeding [3, 7, 12, 78, 85, 90]. As a result, three of the four gorilla subspecies are classified as critically endangered, with the fourth subspecies being classified as endangered [80].

Mountain gorillas are arguably the most intensively studied of all gorilla subspecies. They occur in two isolated populations: one in the mountain ranges of the Virunga Massif, straddling the borders of Uganda, Rwanda and the Democratic Republic of Congo and another in the Bwindi Impenetrable National Park in Uganda [32]. Thanks to conservation efforts, the Virunga mountain gorillas, which experienced a population low of possibly less than 250 individuals in the 1980s [32], have now recovered to around 600 individuals [35]. Together with ca. 400 Bwindi mountain gorillas [36], an estimated 1000 mountain gorilla individuals are currently remaining. The recent population growth experienced by the Virunga Massif led to the declassification of mountain gorillas from critically endangered to endangered [80] —an example of a successful species conservation program.

Geographically and genetically close to the mountain gorillas are the less well known and little studied Grauer’s gorillas, currently facing anthropogenic threats from poaching, agricultural development and illegal mining [63]. Formerly occupying a larger geographic range, Grauer’s gorilla population has declined by up to 80% in the last two decades [63]. They are restricted to fragmented forest patches in the eastern parts of the Democratic Republic of Congo, a region affected by armed conflict, making the ongoing conservation efforts challenging [63]. As a consequence of these events in the last few decades, Grauer’s gorillas have experienced decline in genetic diversity, increase in inbreeding and frequency of deleterious mutations [85].

Western lowland gorillas are found in fragmented patches within the forests of West and Central Africa (Fig. 1A). Despite their relatively large population size compared to the eastern species, they have been severely affected by disease outbreaks, in particular Ebola [7, 12]. The Cross River subspecies is restricted to the forested hills and mountains of the Cameroon-Nigeria border region and has been subject to rapid habitat loss [6]. A 2014 survey estimated that less than 250 mature cross-river gorilla individuals were left in the wild [6] and genetic and recent genomic analyses showed that the population has experienced rapid decline and inbreeding in the last 100–200 years [3, 78].

Recently, whole genome sequencing data was produced for all gorilla subspecies, including both mountain gorilla populations, Bwindi and Virung [60]. In particular, the genomes of Bwindi mountain gorillas have not yet been analyzed in a comparative framework. Levering the now available comprehensive dataset of 53 resequenced genomes (Table S1) allowed us to revisit gorilla evolutionary history, and crucial conservation-related genomic indicators such as genetic diversity, inbreeding, genetic load, and to identify markers of local adaptation across the recognized gorilla subspecies. We aim not only to enhance ongoing and future gorilla conservation initiatives by identifying the genomic repercussions of population threats at fine-scale resolution, but also to shed light on the genetic consequences for species confronting similar threats as gorillas today.

## Results

### The dynamic demographic history of gorillas

Principal component analysis on all autosomal genetic variation shows that the gorilla subspecies are genetically distinct, with a close relationship between the Cross River and western gorillas (Fig. 1B), although a stronger distinction between these subspecies has been reported recently using a larger Cross River gorilla dataset [3]. We also find that the Bwindi and Virunga gorillas separate from each other along the first principal component, confirming their genetic uniqueness, as previously inferred from microsatellite markers [72]. To complement the genetic structure analyses inferred from nuclear data, we assembled and aligned the mitochondrial genome from all samples. Although the eastern and western gorilla lineage are monophyletic, subsequent splits within the two species are only partially resolved (Fig. 1D). Mitochondrially, the Cross River individual is nested within the other western lowland gorillas. All Grauer’s gorillas form a monophyletic sister group to a paraphyletic clade consisting of the Bwindi and Virunga mountain gorillas.

Although low in numbers today, gorilla population size throughout their deep evolutionary history was likely at least an order of magnitude larger [90]. Understanding the long-term history of species and populations is relevant for evaluating the genomic consequences of recent population declines. Following rapid declines, populations with low long-term population sizes often show lower genetic load than populations that declined from historically large populations [86]. All gorilla populations have suffered effective population size reductions in the last few decades [3, 85, 90], hence having accurate knowledge about their long-term population history is important to understand the severity of recent genetic changes. We used a high quality gorilla reference genome and high coverage re-sequenced genomes (> 25X) to estimate effective population size changes between 10,000 and ca. two million years ago using the recently developed beta Pairwise Sequentially Markovian Coalescent (beta-PSMC) based inference method [49, 50]. Our results largely agree with those from previous studies suggesting a large ancestral effective population size of around 40,000 individuals one million years ago, followed by a continuous population decline starting around 200,000 years ago (Fig. 1C). The observed population decline is especially pronounced in eastern gorillas, which started diverging from western gorillas around 150,000 years ago and have remained at low population size since. Whereas previous PSMC analyses suggested a population size increase in the western gorilla species circa 40,000 years ago [65, 90], our beta-PSMC results suggest this population size increase, if any, was small and rapidly followed by further population decline. All eastern gorilla subspecies genomes follow the same population size trajectory, suggesting that any differences among them arose in the past few thousand years, a time range outside of the scope of beta-PSMC inferences (Fig. 1C). Finally, we find that the Cross River gorillas started experiencing a more pronounced decline than the western lowland gorillas around 80,000 years ago, and continued declining to the present-day level that is intermediate to that of western lowland and eastern gorillas.

**Fig. 2:**
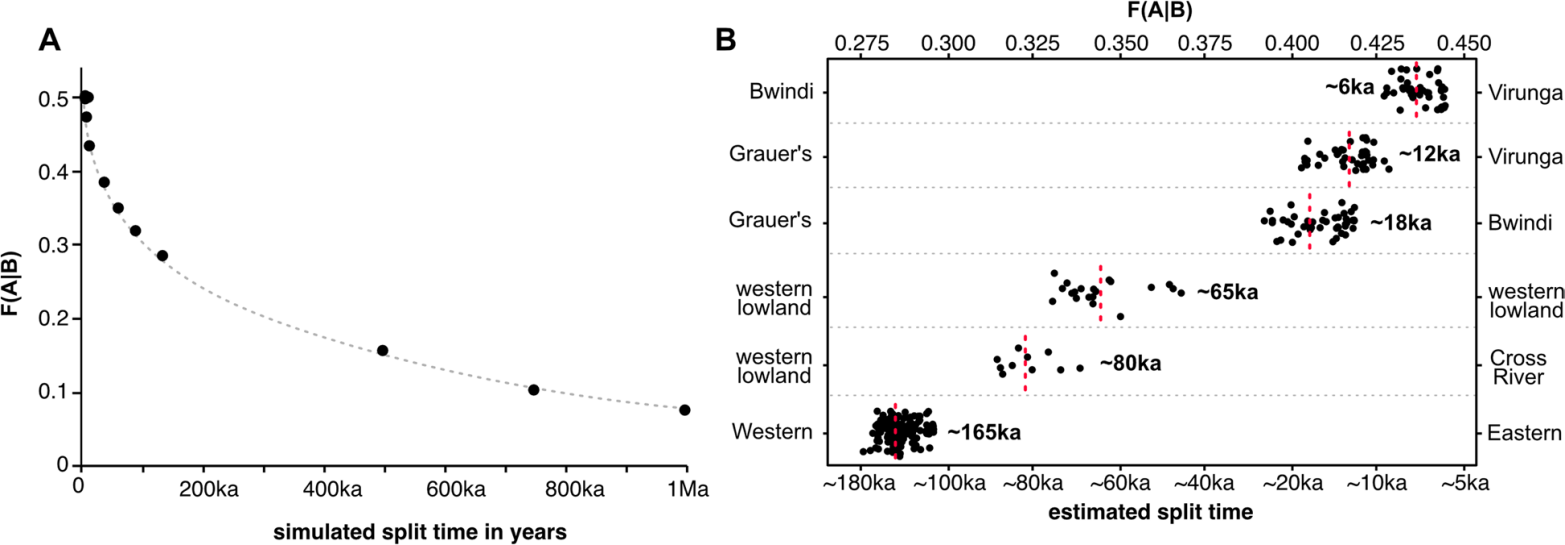
**A** F(A|B) statistic from simulations in msprime. **B** Pairwise F(A|B) statistics for all gorilla genomes. Red dotted line corresponds to estimated split time based on the average across all pairwise comparisons. Each subspecies and both mountain gorilla populations are tested separately, except when combined into eastern and western species

Estimates of the divergence times between the gorilla subspecies from previous studies vary widely, depending on the used generation time, mutation rate and modeled extent and timing of secondary gene flow. For instance, the split time between western and eastern gorillas has been estimated in the range from 100,000 to over 1.5 million years ago, and divergence time estimates between the gorilla subspecies range between 17,800–454,000 years ago for the western lowland—Cross River gorilla and 10,000–20,000 years ago for the Grauer’s—mountain gorilla splits [65, 74, 77, 72, 60, 90]. Leveraging the available high coverage genomes, we used the F(A|B) statistic to estimate population divergence times, defined as the time when significant gene flow most likely ceased to exist between the ancestral populations [28]. The F(A|B) statistic is retrieved by calculating the fraction of sites in which a randomly sampled allele in an individual from population A carries a derived allele in a site that is heterozygous in an individual belonging to population B [28]. To calibrate the F(A|B) statistic to the population size history of the gorillas, we used msprime 1.0 [5] to simulate the population size changes, as inferred from the beta-PSMC trajectory [5]. We then obtained empirical values for the expected decay of F(A|B) and the corresponding divergence times from the simulated output (Fig. 2A). We find that F(western gorilla|eastern gorilla) averages ∼0.28 (Fig. 2B), meaning that western gorillas carry the derived allele in around 28% of the heterozygous sites detected in eastern gorillas. From the simulation of the expected distribution pattern of F(western gorilla|eastern gorilla) given their population history, a mutation rate (µ) of 1.8 × 10^−9^ per generation [8], and a generation time of 20 years [46], we estimate that gene-flow between the two species stopped circa 150,000–180,000 years ago. Based on the F(A|B) statistic, the deepest sub-species divergence is between the western lowland and Cross River gorillas, which we estimate at around 80,000 years ago. We however note that deep divergence times were also recovered between pairs of western lowland gorillas, with some estimates being older than divergence times obtained for Cross River—western lowland gorilla pairs. The divergence between the eastern gorilla subspecies is estimated at much more recent times, between 10,000–20,000 years ago for the Grauer’s—mountain gorilla split and 5,000–10,000 years ago for the Virunga—Bwindi split. It is noteworthy that we obtain a younger split time for Grauer’s—Virunga mountain gorilla comparison (12,000 years ago) than for Grauer’s—Bwindi mountain gorilla comparison (18,000 years ago), suggesting that Grauer’s gorillas are genetically closer to Virunga mountain gorillas than they are to Bwindi mountain gorillas.

### Gene flow events among the gorilla subspecies

We investigated patterns of secondary gene flow among the gorilla subspecies by estimating the amount of derived allele sharing in a pairwise manner (with D and f4 statistics) across all autosomes, using a genome-wide alignment of the human, orangutan and gibbon to infer the ancestral and derived states of the alleles. Although counterintuitive when considering the current distribution ranges (Fig. 1A), we observe elevated allele sharing (Z-score > 7) between the Cross River and Grauer’s gorillas, with an average D of around 0.035 (Table S2). A similar result was reported recently in a study with additional Cross River gorilla genomes and is hence not a spurious signal produced by a single aberrant genome [3]. In addition, we also observe a strong pattern of allele sharing (D ≈ 0.10) between the Virunga mountain gorillas and Grauer’s gorillas (Table S2).

**Fig. 3:**
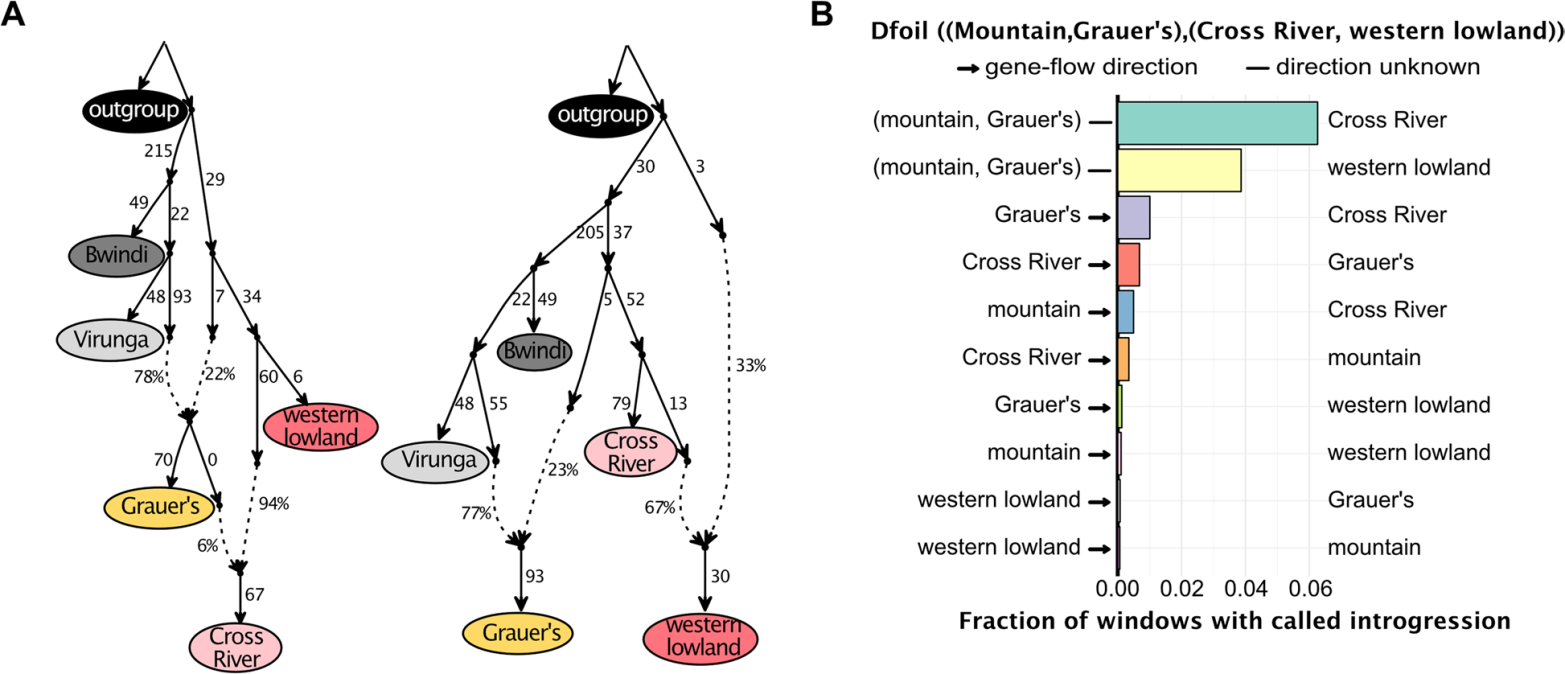
**A** Two qpGraph models without outlier D and f4 statistics showing two admixture events. Numbers next to the arrows correspond to the relative amount of drift on that branch. Percentages next to the dotted lines correspond to the best model fit for the admixture fraction of each population. **B** Dfoil results, showing access allele sharing between the different gorilla populations. Both eastern gorilla subspecies (mountain and Grauer’s gorillas) show a signal of recent admixture with western gorillas, with the strongest signal observed between the eastern and Cross-river gorillas (top bar). Additional bi-drectional gene-flow is observed between the Grauer’s and Cross river gorillas (3rd and 4th bar from the top)

**Fig. 4:**
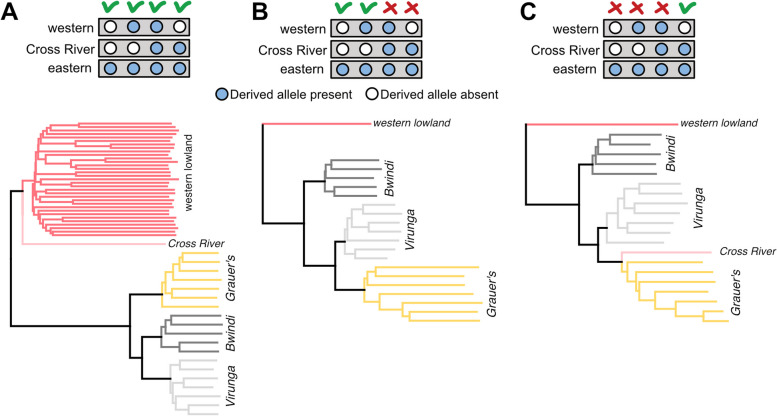
**A** Neighbour joining tree using all autosomal SNPs. **B** Neighbour joining tree removing SNPs with the derived allele present in both eastern and Cross River gorillas. **C** Neighbour joining tree only including SNPs with the derived allele present in both Cross River and eastern-gorillas. Inserts above the genetic trees depict which SNPs have been included or excluded for each of the three phylogenies

We used qpGraphs to model the possible demographic histories fitting the observed D and f4 statistics (Fig. 3). We iteratively tested all possible phylogenetic combinations of all considered gorilla subspecies (treating Virunga and Bwindi as different populations). None of the graph models with less than two admixture events provided a good fit to the data, thus ruling out a simple tree-like population history. In contrast, two different graph models with two admixture events provided a perfect fit (Table S3), explaining all D and *f4*-statistic combinations without outliers (Fig. 3). In both these models, Bwindi mountain gorillas are sister to a clade containing Grauer’s gorillas and Virungas mountain gorillas, contradicting the currently accepted phylogenetic relationship of Bwindi and Virunga mountain gorillas being each other's sister groups (Fig. 4A). In both models, Grauer’s gorillas and the Cross River show an excess of shared genetic ancestry compared to the other gorilla subspecies. In the first model, Grauer’s gorillas are closely related to the Virunga gorillas but have received a significant amount of gene flow (22%) from an ancestral western gorilla population and later contributed 6% of ancestry to what is today the Cross River gorillas. In the second model, Cross River and western lowland gorillas are deeply divergent from each other, with Grauer’s gorillas receiving 23% of their ancestry from a population related to the Cross River gorillas (with the other 77% being Virunga ancestry). According to this model, the western lowland gorillas and Cross River gorillas experienced significant amounts of gene flow more recently, forming the present-day western lowland population.

We additionally used Dfoil, which makes use of D-statistics calculated in four combinations of a symmetrical four taxon topology and an outgroup, to polarize allele sharing patterns, and infer directionality of gene flow [61]. When testing the topology ((mountain,Grauer’s),(Cross River,western lowland)), we found the strongest signal of gene flow between the ancestor of the eastern lineage and Cross River, followed by the eastern lineage and western lowland gorillas (Fig. 3B). We also found support for additional, bi-directional introgression between Grauer’s and Cross River gorillas, and weak signals between the mountain gorillas and the western lineages. The results were qualitatively similar when analyzing Bwindi and Virunga mountain gorilla populations separately (Table S4), and further support a scenario in which Grauer’s gorilla received alleles from the Cross River population, possibly altering the phylogenetic relationships among eastern gorilla populations. Although we caution that stochastic processes such as incomplete lineage sorting may also be at play, all these inferences point to a complex demographic history of gorillas, highlighting the repeated role of introgression throughout their evolutionary history. In particular, the introgression between Cross River and Grauer's gorillas must have occurred after the latter diverged from mountain gorillas 10,000–20,000 years ago and hence rather recently.

To gain a better understanding of the timing of introgression between Cross River and Grauer’s gorillas, we calculated Fd, fdM and dF statistics, measures specifically developed to investigate patterns of introgression in windows across the genome [51, 53, 62]. We then identified all windows in the top 2% of highest introgression signal in all three statistics and merged overlapping windows. The average length of these putatively introgressed windows was 202 Kb (± SD 120 Kb). Based on the average length of introgressed windows, we calibrated the time of admixture using previous estimates obtained from Neandertal and human gene flow. The average size of introgressed Neandertal fragments in humans is 65.69 Kb-88.70 Kb [14] and admixture between these two lineages has been estimated at 47,000–65,000 years ago. Assuming an exponential decay of fragment size over time, the average fragment length of the putatively introgressed windows in gorillas thus corresponds to an admixture event dating to roughly 9,000–12,500 years ago. This independent estimate corresponds well with the timing based on phylogenetic inferences, as it is more recent than the estimated Grauer’s—mountain gorilla divergence of 15-20 ka (Fig. 2B).

To investigate if gene flow has an affect on phylogenetic inferences, we constructed neighbor joining trees based on different genomic regions: i) using all autosomal SNPs (Fig. 4A), ii) excluding sites with the derived allele present in both Cross River and eastern gorilla genomes (Fig. 4B), and iii) using SNPs with the derived allele present among both Cross River and eastern gorillas, but not among western lowland gorillas (Fig. 4C). A phylogeny based on all SNPs groups the Bwindi and Virunga mountain gorilla populations as a sister clade to Grauer’s gorillas (Fig. 4A). This phylogeny is in accordance with the patterns of the PCA analysis (Fig. 1A), and agrees with the generally accepted relationships among gorilla subspecies and populations [60]. However, when we excluded sites where the derived allele was present in both Cross River and eastern gorillas, which removes any signal caused by introgression between the two, Grauer’s gorillas are phylogenetically closer to the Virunga mountain gorillas than the Virunga population is to Bwindi mountain gorillas (Fig. 4B). A similar topology was recovered using only SNPs with shared derived variation among Cross River and eastern gorillas, with this analysis additionally grouping the Cross River gorilla as a sister lineage to Grauer’s. This supports that alleles have been exchanged primarily between Cross River and Grauer’s gorillas. These phylogenies additionally suggest that Grauer’s gorilla share a common ancestor with Virunga mountain gorillas that post-dates the split between the Virunga and Bwindi populations. Grauer’s gorillas thus appear more divergent to both the Bwindi and Virunga in the full SNP dataset due to the presence of alleles that introgressed from the genetically divergent Cross River gorilla. Although our methods are not suitable to formally test for gene flow between Bwindi and Virunga mountain gorilla populations, the mitochondrial phylogeny indicates that their sister relationship was likely reinforced by gene flow that postdates the separation between Virunga and Grauer’s gorillas.

### Comparative gorilla genetic diversity

**Fig. 5:**
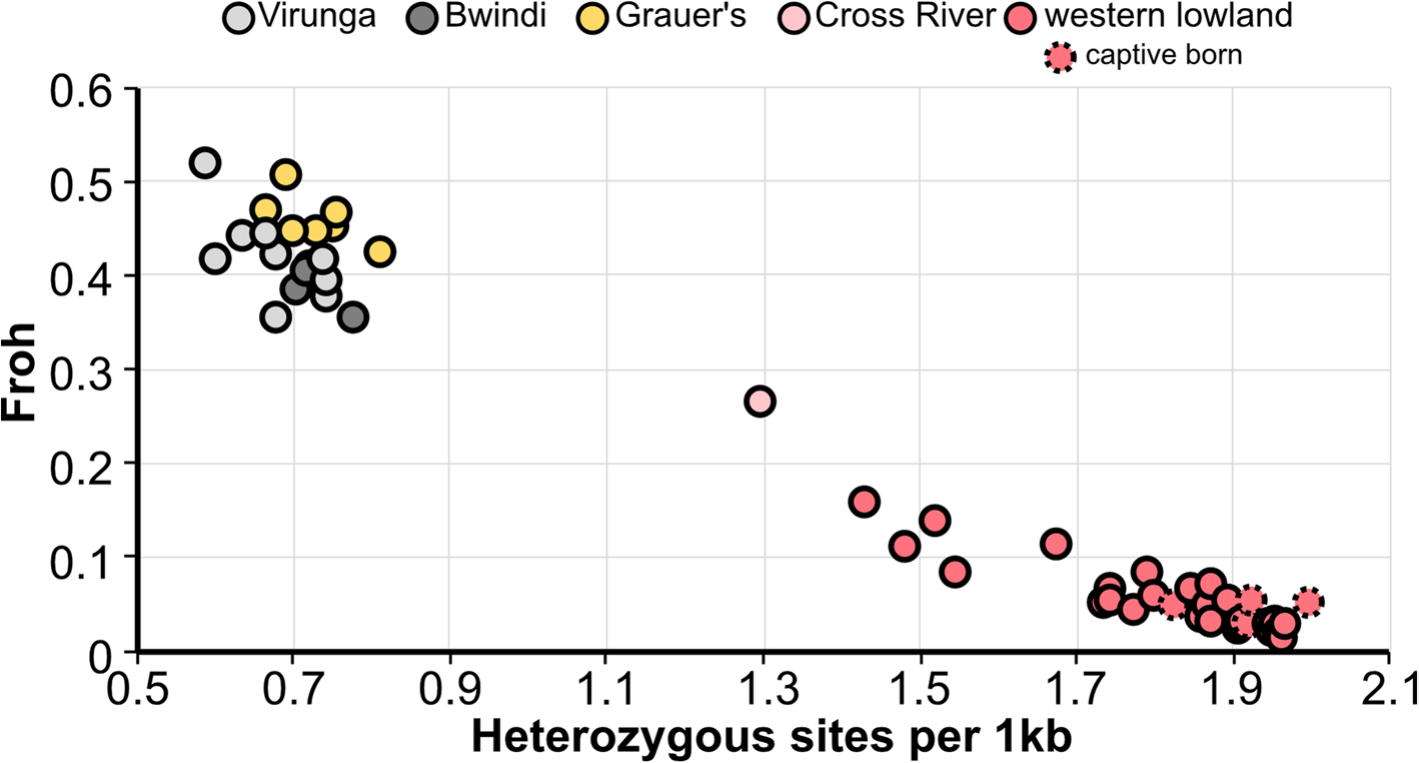
Autosomal heterozygosity (x-axis) and genomic inbreeding, displayed as the fraction of the genome in runs of homozygosity exceeding 100 kb (y-axis)

After variant calling and filtering across all genomes, we identified more than 22 million single nucleotide polymorphisms (SNPs) across all individuals included in the dataset, with 15.2 million SNPs at frequency > 5%. Of these variants, 35% were found in both gorilla species, 51% were found only in western gorillas and 14.3% of variants were unique to eastern gorillas. Similar patterns of diversity, with more diversity in western compared to the eastern species, were found for indels. Among indels, 59.0% were unique to western gorillas and only 13.9% were unique to eastern gorillas. We estimated conservation-relevant genomic parameters by measuring genome-wide autosomal heterozygosity and the number and length of long runs of homozygosity in each gorilla genome. Eastern gorilla heterozygosity ranged between 0.55–0.80 per thousand base pairs (kb), which is threefold lower than the 1.4–2.0 per kb observed in the western lowland gorillas (Fig. 5). The difference is driven by the large fraction of the eastern gorilla genomes, between 35 and 50%, in long runs (> 100 kb) of complete homozygosity, whereas this fraction is below 10% in most western lowland gorilla genomes (Fig. 5). Long runs of homozygosity are a measure of the relatedness between mating pairs in past generations, and thus roughly reflect recent effective population size. Among eastern gorillas, the Grauer’s gorillas showed the highest genome fraction in runs of homozygosity, despite being the largest extant eastern gorilla population. This suggests that they have experienced a bottleneck after their split from the Virunga mountain gorillas, in line with findings that recent drastic population declines have led to reduction in genetic diversity and increase in inbreeding in this subspecies [85]. Among western lowland gorillas, several genomes from wild born individuals also contained long runs of homozygosity, encompassing up to 18% of their genome. It is possible that some individuals originated from isolated populations, however it could also be an effect of the western gorilla social structure, with stronger male reproductive skew compared to eastern gorillas [10, 70]. Measures of heterozygosity and runs of homozygosity in the Cross River genome are intermediate to eastern and western lowland gorillas, confirming that the present-day small population size has already left its mark on the genome. We observe that the fraction of the genome in runs of homozygosity is on average 17% higher in the Virunga mountain gorillas compared to Bwindi. Since both Bwindi and Virunga populations are currently similar in size, counting 400 and 600 individuals, respectively, higher inbreeding in Virunga gorillas is likely the result of the population bottleneck in the 1960s [4, 31]. We also measured the ratio between heterozygosity on the X-chromosome and the autosomes. We observe values between 0.34 and 0.55 in all gorilla subspecies, with the exception of the Virunga mountain gorillas, for which the ratio is on average 0.24. Under neutrality and random mating this ratio is expected to be 0.75, as the Ne for X-chromosome and autosomes corresponds to a 3:4 ratio. The lower-than-expected ratio in gorillas is likely a reflection of their social structures, with high male reproductive skew and sex-specific differences in dispersal distances [18, 71]. Because the sex chromosomes and the autosomes have different effective population sizes, a population bottleneck that affects males and females equally will reduce diversity more on the X chromosome than on the autosomes [64]. Thus, the bottleneck experienced by the Virunga mountain gorillas also seems to have left a strong signal on the X chromosome diversity.

Finally, we investigated autosomal regions with particularly high or low genetic diversity across all gorilla individuals. We find 42 regions > 50 kb in length that have five times higher heterozygosity in all gorillas compared to the genome wide average. These regions contained 44 genes, of which seven are coding for cell surface proteins essential for the adaptive immune system (major histocompatibility complex proteins) and five are coding for pregnancy specific beta-1-glycoproteins, which are vital in fetal development and functions primarily as immunomodulators protecting the growing fetus (Table S5). High genetic diversity is usually mediated either through heterozygote advantage or frequency-dependent selection, mechanisms frequently described for immune-related genes. We further identified 85 autosomal regions with less than one fifth of the genome wide heterozygosity in all gorillas, overlapping 54 coding genes. Although, we did not find significantly over-represented gene ontology categories among these genes, most were associated with primary cell function pathways, such as histone methyltransferase (KMT2D), intracellular pumps (ATP2A1), water channels (AQP6) and proteolysis (PRSS36), suggesting that these gene regions are under strong purifying selection (Table S5).

### Mutational load

Along with genetic diversity parameters, we investigated putative deleterious genetic variation across the gorilla genomes that could contribute to effective genomics-informed conservation strategies. As we did not have population level data for the Cross River gorillas, we omitted this subspecies from these analyses. We used SIFT, an algorithm that predicts the effects of amino acid substitutions on protein functions based on protein conservation with homologous sequences and the severity of the amino acid change, to distinguish between likely deleterious and tolerated genetic variants. To increase SIFT accuracy, we lifted over the gorilla variants to the human genome and ran SIFT using the extensive human protein annotation database. For each genome, we counted the number of coding genes containing genetic variants predicted to have a high impact on the protein function. We find on average 3,344 genes affected by either a frameshift, high impact missense or stop-loss/gain mutation in the western gorillas. In contrast, the eastern gorillas have on average fewer genes affected by deleterious variants: 3,026 and 3,015 in Virunga and Bwindi mountain gorillas, respectively, and 2,976 on average in Grauer’s gorillas. The number of deleterious alleles in the gorilla subspecies is roughly inversely related to the genome fraction in runs of homozygosity, which can be explained by genetic purging [26]. Inbreeding, as a consequence of small population size, causes alleles to be more frequently exposed in a homozygous state, and thus strongly deleterious alleles are more often removed by selection in an inbred population even if they are recessive [22, 86]. It is noteworthy that despite the considerably larger current population size, Grauer’s gorillas carry fewer deleterious variants than mountain gorillas. This pattern is in line with the notion that Grauer’s gorillas likely experienced a population bottleneck after their divergence from the Virunga gorillas [81].

### Local adaptation on the genomic level

**Fig. 6:**
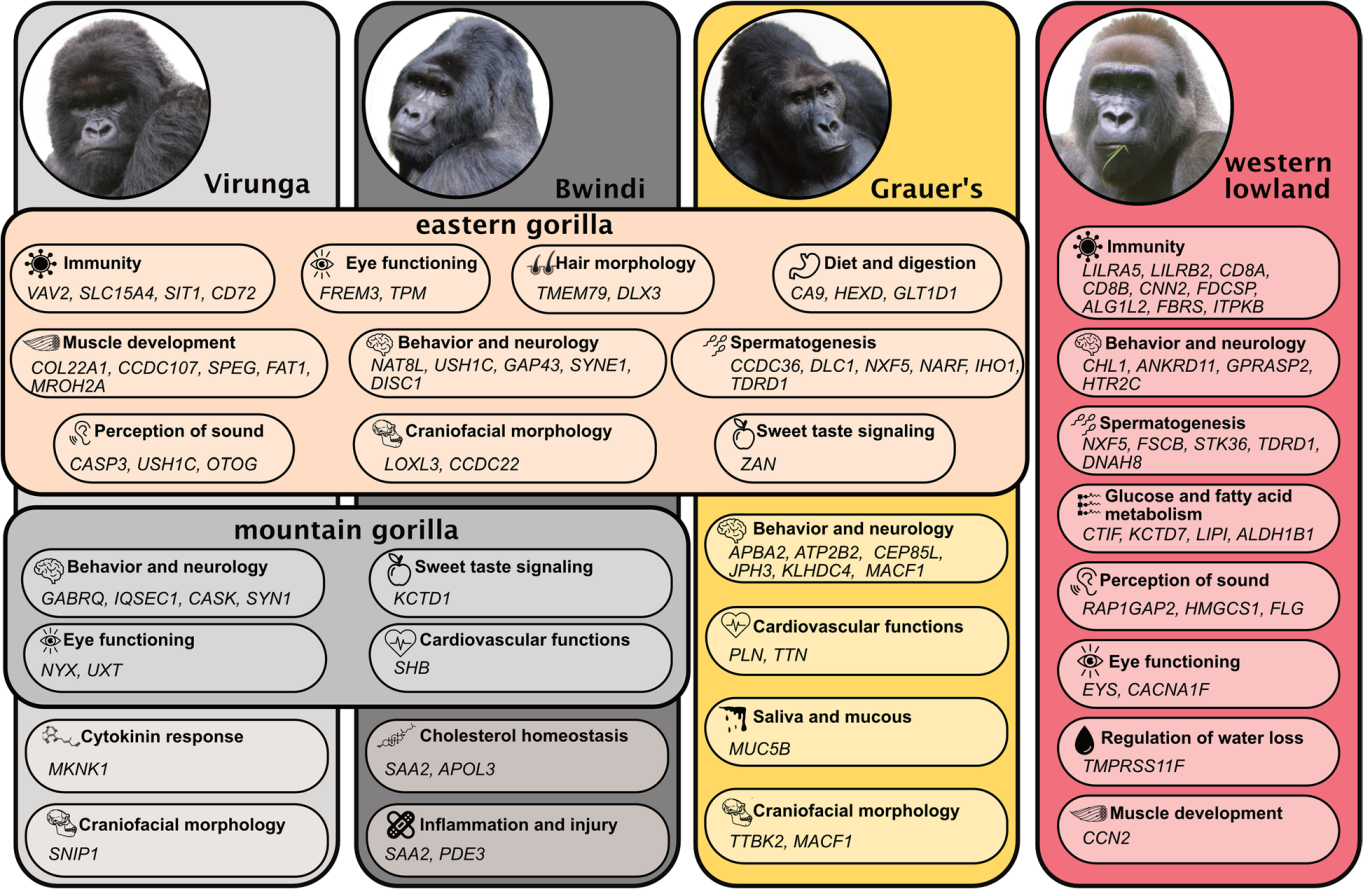
Examples of main functional categories and associated genes identified as putatively important for local adaptation among the gorilla (sub)species and populations. For a complete list of genes, see Table S10. Photos by Damien Caillaud

In addition to genetic diversity, local adaptation is crucial in endangered species, as the presence of adaptive alleles not only ensures their long-term survival but can also affect the success of conservation actions, e.g. translocation of individuals for genetic rescue. We investigated patterns of local adaptation across the gorilla subspecies using two independent strategies: Cross-Population Extended Haplotype Homozygosity (XP-EHH) selection statistics [45] and identification of protein sequences unique for each subspecies (Fig. 6).

XP-EHH performs a genome-wide haplotype-based selection scan in windows across the genome [45]. The XP-EHH scores were calculated in a pairwise manner for all western versus all eastern gorillas and for all Grauer’s versus all mountain gorillas. A comparison between the Bwindi-Virunga populations was excluded due to paucity of divergent SNPs and all comparisons with the Cross River gorilla were excluded due to the small sample size. We identified all genes within 10,000 base pairs from the focal windows showing a strong signal of selection (log *P*-value > 5) (Table S6). In total, we identified 37 annotated coding genes specifically under selection in eastern gorillas and 109 such genes in western gorillas. The almost threefold higher number of genes under selection in western gorillas is possibly a reflection of their larger historical and current population size, and thus stronger effect of selection compared to the eastern gorilla species.

As a second approach, we identified all protein sequences that are fixed for unique variants in each of the gorilla (sub)species and populations, a strategy recently used to study adaptive evolution in woolly mammoths [17]. The underlying reasoning is that coding variants that are important for adaptation in the local environment reach fixation but remain absent or at low frequency in populations where they do not convey adaptive benefits. First, out of the 19,355 annotated genes, we detected 4,480 that were conserved, with no coding variation among the protein sequence in all gorilla genomes analyzed here (Table S7). A gene ontology enrichment of these conserved genes revealed several overrepresented terms, mostly related to basic metabolic functions (Table S8). We however identified 41 protein sequences unique to western gorillas and 167 unique to eastern gorillas (Table S9). The lower number of conserved unique genes in western versus eastern gorillas is a consequence of the higher genetic diversity in western gorillas and a larger sample size (33 western vs 22 eastern genomes), which allows lower frequency variants to be detected in the western species.

Both approaches revealed a set of gene functional gene categories that are in agreement with each other. When comparing eastern and western gorillas, XP-EHH and protein sequence conservation approach both detected genes involved in immunity, diet and digestion, behavior and neurological functions (Fig. 6, Table S10). It is noteworthy that the specific sets of genes involved in these functions were different between the species (Fig. 6), in line with different diets (e.g. higher fruit consumption in western gorillas, [21, 37, 56, 68]), social organization [69], and ecological differences in their habitat, which likely also involve differences in pathogens. The two approaches also identified genes associated with the perception of sound, function of the eye, and genes involved in skeletal and cardiac muscle cells, and muscle weakness and fatigue in eastern gorillas, the larger and more muscular subspecies. Each gorilla species contained a unique fixed set of protein sequences involved in spermatogenesis and hence reproductive processes (Fig. 6, Table S10). 

In the comparison between Grauer’s and mountain gorillas, we uncovered genes involved in skeletal and cardiac muscle strength, brain and neurological functions, diet, and digestion (Fig. 6, Table S10). The two eastern subspecies differ from each other in social organization, markedly in the proportion of multi-male groups [70] and several of the detected genes are proposed to be involved in social behavior and aggression, suggesting that behavioral functions might be under selection. Grauer’s gorillas occupy lower altitudes than mountain gorillas and the diets between the two subspecies differ, specifically in the proportion of consumed fruits and the diversity of dietary items [33, 57]. Genes under selection in mountain gorillas are involved in sweet taste signaling, lipid catabolic processes, and regulation of insulin secretion. We caution, however, that due to the levels of inbreeding in eastern gorillas, the used selection statistics can be biased [34]. Additionally, the low population size of the eastern gorillas results in a high amount of drift, which may create patterns that could be wrongly interpreted as signals of selection [44].

Finally, the comparison between Bwindi and Virunga mountain gorilla populations uncovered unique protein sequences in Bwindi, which play an important role in cholesterol homeostasis and mediate platelet aggregation (Fig. 6), which is involved in high altitude adaptation. Protein sequences unique to Virunga were involved in cytokine and environmental stress response and craniofacial morphology.

## Discussion

**Fig. 7:**
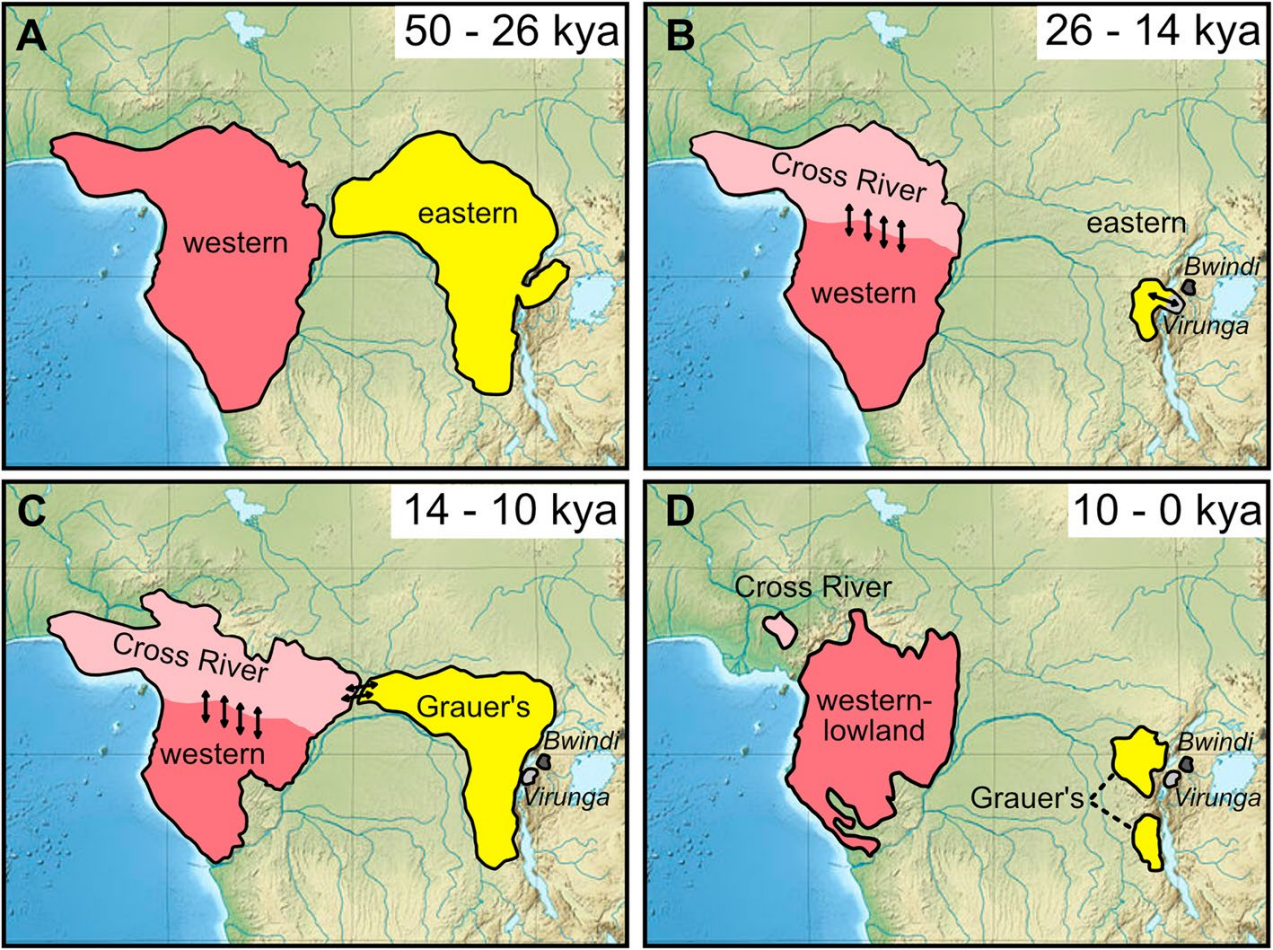
Hypothesized gorilla population dynamics over the course of the last 50,000 years. Ranges and geographical locations in panels A-C are rough approximations, whereas D is based on present-day IUCN species ranges, same as Fig. 1A. **A** Western and eastern gorillas split prior to the Last Glacial Maximum (LGM). **B** Global cooling during LGM led to forest contractions and reduction of population sizes of both western and eastern gorillas. Bwindi becomes separated from other eastern gorillas. **C** During the African humid period, eastern populations expand westwards and separate into Grauer’s gorillas and the Virunga mountain gorilla population. Gene flow occurs between Cross River ancestors and Grauer’s gorillas. **D** Aridification in east Africa, leading to reduction in population size

Despite decades of genetic studies on gorillas, much of their evolutionary history remains a mystery. We detected multiple signals indicative of introgression between gorilla subspecies, which together with recently reported admixture from a possible unsampled “ghost” population [60] show that gene flow was likely common throughout gorilla evolution. The inclusion of whole genome data from Bwindi mountain gorillas allowed us to uncover novel aspects of the gorilla evolutionary history. We detected a strong genetic similarity between Virunga mountain gorillas and Grauer’s gorillas to the exclusion of the Bwindi mountain gorilla population, and obtained indications of gene flow between Cross River and Grauer’s gorillas, likely to the exclusion of all mountain gorilla populations. The here reported evolutionary relationships among eastern gorillas are in disagreement with previous studies [72, 90] and the inferred Cross River—Grauer’s gorilla gene flow appears contrary to expectations based on present-day geographic distributions. While acknowledging potential biases in gene flow statistics stemming from factors such as incomplete lineage sorting, variations in mutation rates, ancestral population structures and biases in batch effects and reference mapping [30, 52, 82], we hypothesize the following geographic scenario that is consistent with the observed test statistics: Prior to the Last Glacial Maximum (LGM), 50,000–26,000 years ago, eastern gorillas were likely distributed throughout Eastern Africa, reminiscent of the current distribution range of eastern chimpanzees (*Pan troglodytes schweinfurthii*) (Fig. 7A). During the LGM, 26,000–20,000 years ago, rainforests in this region of Africa retreated in favor of afromontane and savannah landscapes as temperatures, rainfall, and humidity significantly decreased [11, 24]. During this time, the East African lakes including lake Victoria, lake Albert and lake Edward dried out, and lake Tanganyika shrunk considerably [11, 24, 58]. This dry period possibly led to the separation of the Virunga and Bwindi mountain gorilla populations (Fig. 7B). Around 14,500 years ago, with the onset of the African humid period, lakes started to re-appear [24] and development of forest vegetation around the African Great Lakes created an interconnected environment, allowing forest-dependent species to spread, leading to a marked increase in biodiversity [42]. The increased humidity during this period caused a significant expansion of East African rainforest and likely allowed a westwards expansion of a small gorilla population from the Virunga region into the region that is today occupied by Grauer’s gorillas (Fig. 7). This expansion was associated with an initial bottleneck, which is also visible in the structure of mitochondrial haplotype networks of Grauer’s gorillas [84]. Simultaneously, a population genetically most similar to contemporary Cross River gorillas expanded eastwards from its range in western Africa, where it exchanged genes with the westwards dispersal front from the Virungas at the edge of their distribution, giving rise to Grauer’s gorillas (Fig. 7C). The African humid period ended about 6,000–5,000 years ago, leading to a decline of forested habitat, with the Sahara becoming barren and being claimed by sand [16, 55]. Possibly it was during this time period that Grauer’s, Virunga and Bwindi populations became completely isolated and restricted to their present-day ranges, with further recent population declines from anthropogenic pressures (Fig. 7D).

Despite the relatively recent divergence between the gorilla (sub)species, we identified genes under selection in each species as well as a set of protein sequences unique to each subspecies and population, encoding for various traits. Thus, despite indications of recent gene flow (< 20 ka) between the two species, they each exhibit a signature of local adaptation and should thus continue to be treated as evolutionary distinct units. Similar to previously reported genetic purging in the Virunga gorillas [90] we also detect purging of deleterious variants in the Bwindi mountain gorilla population. In addition, despite their low population numbers, genetic diversity in the eastern gorillas is similar to that of non-African humans and we hypothesize that the relatively low levels of genetic load and present-day genetic diversity therefore do not necessarily pose an immediate threat to the short-term survival of eastern gorillas. However, the signals of local adaptation in for example immune-genes, suggest that preserving the unique genetic diversity in each of the gorilla subspecies could be of importance for their long-term survival.

Finally, despite limited sampling, we observe deep genetic divergences between several western lowland gorilla individuals. This suggests that a large fraction of diversity and population structure in this species remains undiscovered, with unstudied western lowland gorilla populations likely carrying unique genetic variants. Such populations might be especially important for conservation, as they can for instance contain variants associated with an increased chance of survival during disease outbreaks [20]. Despite comprehensive research efforts, gaps in our understanding of one of our closest living human relatives, the gorillas, remain. Ensuring their preservation to the greatest possible extent will not only safeguard these species into the future, but also facilitate the exploration of crucial questions pertaining to our own evolutionary history.

## Methods

### Genome processing

FASTQ data for all previously published high-coverage (> 10X) gorilla genomes was obtained from ENA (Table S1). Next, we removed sequencing adapters and subsequently bases at the start and end of the reads were trimmed if falling below a quality score of 20 using FASTP [13]. Filtered reads were then mapped against a composite reference, consisting of the latest version of western-lowland gorilla reference genome (GCA_008122165.1) and a gorilla Y-chromosome (GCA_015021865.1) using bwa-mem on default settings, including readgroups and marking shorter split hits as secondary [48]. After mapping, alignments around indels were improved using GATKv3.7-indelRealigner [54] and we removed sequence duplicate reads using Picard v2.27.5 (https://broadinstitute.github.io/picard/). Next, for each individual gorilla genome we calculated the minor allele frequency on the mitogenome for sites where more than one allele is supported among the reads using samtools v1.15 mpileup and a custom python script [47]. Such sites are expected at low frequency (< 0.1%) due to sequencing errors or heteroplasmy. In contrast, when significant (cross)contamination of a sample has occurred the minor allele frequency at variable mitogenomic sites would show a relatively high frequency [83]. Two genomes (ENA IDs SAMEA2697043 and SAMEA2697040) showed a minor allele frequency on the mitogenomes above 2% and were conservatively removed from all further analysis, resulting in a total dataset of 53 gorilla genomes, comprising all 4 recognised subspecies as well as multiple genomes from both isolated mountain gorilla populations (Bwindi and Virunga).

### Single nucleotide polymorphisms and indel calling

We called single nucleotide polymorphisms (SNPs) and indels using GATKv4.2 HaplotypeCaller on default parameters. Raw variant calls were subsequently filtered following recommendations of the GATK filtering guidelines using separate filterers for SNPs and indels respectively [87]. Specifically, we used the following GATK filter settings for SNPS: -QD < 2, -FS > 60, -MQ < 40, -SOR > 4, -MQRankSum < -12.5 and -ReadPosRankSum < -8.0. For indels we used the following recommended filtering parameters: -FS > 200, ReadPosRankSum < -20.0, -MQ < 30. Additionally, we removed all SNPs below a quality 30 (phred-scale), those with less than half or more than double the average genome-wide coverage using VCFtools [15] and all heterozygous sites for which the minor allele was only supported by a single read (Table S1). To infer the ancestral state of the sites in the gorilla genome we aligned the human (GCA_000001405.29), orangutan (GCA_002880775.3) and gibbon (GCA_006542625.1) reference genomes to the gorilla reference using minimap2.2.24 with the parameters -ax asm10 to allow for up to ~ 10% of sequence divergence between the alignments. We then set the ancestral allele for all sites where the human, orangutan and gibbon genome showed the same allele.

### Heterozygosity and runs of homozygosity

We estimated heterozygosity in 10 kb windows across each individual genome by generating samtools pileup files [47] for all genomic sites covered by at least 10 reads and subsequently calculating the number of sites for which the allele frequency of the reference and alternative allele where between 10 and 90%. By restricting the analysis to sites of depth 10 and a minor allele frequency of at least 10%, we automatically restricted the identification of heterozygous sites to those for which both alleles are supported by at least two independent sequence reads. In addition, we also excluded sites for which any of the mapped reads suggested the presence of an indel, as such regions can be enriched for mis-alignments [1]. Next, we classified all 10 kb windows with three or fewer heterozygous sites as complete homozygous and merged all adjacent homozygous windows into a bed-file [67]. Merged windows with a size of at least 100 kb were then classified as a run of homozygosity.

### Population structure and demographic history

In order to assess population structure among all our genomes we performed a principal component analysis on all filtered autosomal SNPs using plink v1.9 on default parameters [66]**.** To infer the long-term demographic history of the two gorilla species (western and eastern gorillas), we used the pairwise sequentially Markovian coalescent model (beta-PSMC) [50]. As the beta-PSMC analysis is sensitive to genome sequence depth, we used the five western and eastern gorilla genomes with the highest coverage (all > 25X). We excluded sex chromosomes, repetitive regions as obtained from the NCBI repeatmask track and all sites for which read depth was less than half and above two times the genome-wide average from the analysis. We scaled the output to years using a generation time of 20 years [78] and a mutation rate of 1.8 × 10^−8^ per site per generation [8]. Finally, to infer split time estimates of the ancestral gorilla populations we estimated the probability that at sites where an individual B is heterozygous a second individual A carries the derived allele at a single randomly chosen chromosome (F(A|B) statistic) [28]. This method is based on the assumption that two diverging populations accumulate independent mutations that will not, assuming complete isolation, be present in other populations. By randomly sampling a single allele from the A individual, population size fluctuations in the A lineage do not affect the inference. Thus, we only need to account for genetic drift in the population leading to individual B. To calibrate the F(A|B) to the population size history of the B individual, we simulated the population size trajectory using msprime 1.0 [5] with the inferred trajectory from beta-PSMC as model parameters and obtained the empirical values for the expected decay of F(A|B) and the corresponding split time from the modelled output.

### Mitochondrial assembly and phylogeny

We de novo assembled the mitochondrial genomes for all samples using MitoFinder [2]. First, any remaining adapter sequences were removed with trimmomatic on default settings [9], and MitoFinder was run with the metaspades assembler with default settings on the adapter trimmed reads. For all but two samples we obtained the complete contiguous mitochondrial genome. Two samples (SAMEA3939557 and SAMEA2697039) rendered discontiguous assemblies of two contigs each, which were then aligned to a publicly available gorilla mitogenome (NC_011120.1) and manually concatenated or merged, restoring a complete and near complete (the two contigs were joined by 1091 unknown bases) mitochondrial genome for these samples, respectively. Next, the assemblies were shifted to the same starting position using a custom Python script, and aligned with mafft [43]. Finally, we excluded the hypervariable D-loop, and used PhyML on default settings (HKY85 model) to construct a neighbor joining tree [29].

### Gene-flow

We used Dsuite v0.5 to calculate D and f4 statistics for all possible combinations of samples using the filtered autosomal SNP dataset and human, orangutan, gibbon genome alignments for the ancestral allele inference [51]. Dsuite was also used to calculate Fd, fdM and dF statistics, measures specifically developed to investigate patterns of introgression in windows across the genome. All three introgression statistics were calculated in a pairwise manner using a mountain gorilla genome as population 1, a Grauer’s gorilla genome as population 2, and the Cross-river gorilla genome as population 3, a window size of 20 SNPs and a window stepsize of 10 SNPs, rotating through all combinations of individuals. Next, we converted the filtered autosomal SNP data to eigenstrat format and constructed admixturegraphs, iterating through all possible graph combinations for the five gorilla subspecies/populations (wester, Cross-River, Grauers’s, Virunga and Bwindi) using qpBrute v0.3 [59]. At each iteration, insertion of a new graph node was tested against all existing branches of the graph. In cases in which a node could not be inserted without producing f4 outliers (that is, |Z|≥ 3), all possible admixture combinations were also attempted.

To polarise the D-statistics and infer directionality of gene flow, we used the Dfoil statistic [61]. Dfoil calculates four different D-statistics for different combinations in a symmetrical four taxon topology with an outgroup (((P1,P2),(P3,P4)),Outgroup) and uses the combination of these to identify the donor and recipient lineages, as well as the directionality of gene flow terminal branches. We counted the frequency of the 16 biallelic site patterns (from AAAAA to BBBBA) in sliding windows of 100 kb using a step size of 200 kb across all autosomes with a custom Python script. Next, these windows were analyzed with dfoil.py and summarized with dfoil_analyze.py (https://github.com/jbpease/dfoil). The analysis was run with the human/orangutan/gibbon consensus as outgroup, western lowland as P4, Cross River as P3, Grauer’s as P2 and repeated with either Bwindi, Virunga, or the two joined together as P1. All autosomal neighbor joining trees were constructed using fasta alignments in MEGA11 [76].

### Local adaptation and coding variation among gorillas

To identify signals of recent local adaptation among the gorilla populations we used the XP-EHH statistics, which is based on extended haplotype homozygosity (EHH) statistic as formulated by [73] and implemented in the rehh R package [25]. The XP-EHH selection scan was run across the genome on the filtered SNP dataset using two different population groupings 1) All western gorillas as population A and all eastern gorillas as population B and 2) All Grauer’s gorillas as population A and all Mountain gorillas (Bwindi + Virunga) as population B. In addition to genomic selection signals, we also searched for protein-coding alleles unique and fixed to each of the gorilla subspecies and populations. We separated the SNP dataset into subsets; identifying sites for which all gorilla individuals in the same population are homozygous for the derived allele and all other gorillas homozygous for the ancestral allele. Next, we lifted over all these alleles to the human reference genome GCA_000001405.29 using the human-gorilla minimap2 alignment and a chain file generated using transanno v0.3.0 (https://github.com/informationsea/transanno.git). We then used SIFT to identify allele protein-coding changes among the population specific allele sets and obtained SIFT scores, a measure of how likely these changes are to impact the protein function, for each of the alleles [88].

### Gene ontology enrichment

We conducted all gene ontology (GO) enrichments using GOrilla [19], with the subset of genes of interest as target set and the full set of annotated protein coding genes in the human genome as background set.

### Supplementary Information

**Additional file 1:** **Table S1.** Resequenced gorilla genomes analysed in this study and allele balance of heterozygous sites after mapping. **Table S2.** D and f4-ratio statistics for each pairwise comparison of the gorilla genomes. **Table S3.** qpGraph statistics for the two models in figure 3A. **Table S4.** Dfoil results with different P1 populations. P2-P4 was the same in all analyses: P2 = Grauer's (G), P3 = cross river (CR), P4 = western lowland (WL). The consensus sequence of human/orang-utan/gibbon was used as outgroup. **Table S5.** Genes within low or high genomic diversity regions. **Table S6.** XP-EHH selection statistics for genes with a log *P* value above 5. **Table S7.** Genetic variants across all gorilla genomes per gene. **Table S8.** Gene ontoholgy enrichment of conserved genes among gorillas. **Table S9.** Genetic variants unique to each gorilla (sub)species and the SIFT annotations based on the human genome protein annotation. **Table S10.** Genes under selection and/or with unique protein sequences in each gorilla (sub)species.

## Data Availability

All data used in this study is publicly available on the European Nucleotide Archive under the following project IDs; PRJEB3220, PRJNA189439 and PRJEB12821.
